# Notes and News

**Published:** 1890-01-11

**Authors:** 


### Notes and News.

Collected and Communicated.

A True Philanthropist : Mr. Peter Reid's £100,000.—It has been an open secret for some days past that the donor of £100,000 for the foundation and maintenance, within easy access of London, of a convalescent home for the reception of hospital patients who have arrived at the stage of convalescence was Mr. Peter Reid. This grand offer is another proof of the great generosity of the present age. It is also a proof of the present practical spirit, for in no direction is charity more needed than in the care of patients discharged from our largest hospitals. St. Bartholomew's already has its own convalescent home, and the London and Middlesex have been named as the two institutions most worthy to be helped. Mr. Peter Reid is a governor of St. Thomas's, St. Bartholomew's, and many other hospitals and charitable institutions, in the welfare of which he has long been known to take a serious interest. Mr. Reid is a remarkable man in many ways, and is deservedly one of the most popular of City men. He was until recently a partner in the firm of P. Cozenove and Co., the members of which have always set an example of princely munificence. Commencing life in comparatively humble circumstances, Mr. Reid is reported to have made it a rule of his life to set aside every year one tenth of his income for charitable purposes. His latest and most generous gift is a gratifying proof of the influence members of the medical profession are so often able to exercise in guiding the impulses of the wealthy into useful channels of benevolence. Sir William Savory and Mr. Peter Reid have long been intimate friends, and it is gratifying to be able to recognise the wisdom which has been exercised in the choice of an object to which this magnificent gift could be usefully devoted. Mr. Peter Reid is one of the kindest and best of men, and his many friends will heartily endorse our expression of gratitude to the generous donor of so princely a sum as £100,000.

A child died from influenza last week, and nurses at Charing Cross, Guy's, and the London are down with the epidemic, the serious nature of which we at length begin to recognise. It is amusing to see the boisterous person laugh cheerfully at the new epidemic, and say it may be very modish to have influenza, but that for his part he calls a cold a cold. A week later you meet the same individual in a weak and depressed condition, and he moaningly states that he has had influenza, and had no idea it was like *that.* "Not in the least like a common cold," he confesses; "much more like rheumatic fever. Cold shivers and hot pains for three days ; and it has left me as weak as a rat, and with a raw throat, so that I can't smoke !"

The Earl of Aberdeen is to preside at the dinner at the Hotel Metropôle on January 29 in aid of the East-end Mothers' Home. This deserving charity has lately secured fresh premises in the Commercial-road, and £2,000 is badly needed to furnish and adapt it. Since the opening of the home on December 6, 1884, to April 30, 1889, 545 patients have been registered for admission, 309 have been admitted and discharged well, 59 were attended at their own homes, chiefly because the institution being full at the time there was no room for them, 125 of these were treated during the past year with the most satisfactory results, not one death having occurred from the commencement. The dinner tickets are free, and it is expected that the funds will be all the greater. A large number of ladies will probably be present.

The Sisters of Mercy who manage the Mater Infirmorum Hospital at Belfast have published their annual report. During the twelve months ending October 31st, 1889, 211 patients received treatment in the wards of the hospital. Of these 141 were suffering from medical and 70 from surgical diseases ; 203 were discharged, cured, or improved ; eight died, of whom two were moribund on admission. A considerable number of important surgical operations have been performed with the most satisfactory results. At the extern department 3,085 cases received treatment, and numerous minor operations were performed. The income from all sources, including the balance from last year, amounted to £1,048 11s. 7½d., and the expenditure to £610 3s. 7½d., leaving a balance in hand of £438 8s. The hospital is often overcrowded, so with a balance like the above it seems wrong not to enlarge it.

On Friday, December 27th, a most successful entertainment was arranged for the benefit of the Torbay Hospital, which realised the handsome sum of £33 13s. The waxworks were interspersed with tableaux, music, singing, and recitations; all were fully appreciated, though, as usual in Torquay, the audience was not demonstrative. The company was aristocratic and numerous ; there was a little over-crowding, caused by the company not filling up the seats as they came in. In September there was a concert given, which brought to the funds £20. There is, no doubt, much pleasure given, but the work they entail is great, and falls mostly on one or two persons ; therefore we would say, "Honour to whom honour is due." We may add the entertainment was given in one of the wards which had been turned out for cleaning.

A woman died last week in the Blackfriars-road whose husband searched for four hours and could not find a doctor to attend to her. The woman died from pneumonia, and, as it happened, the delay made no difference ; still, the fact that it took four hours to find a doctor to visit a patient at night was commented on by several of those present at the inquest. Dr. Jones said he should like to add that he thought there ought to be some system of night medical service. He had had people come to his house at night and say they had been after twenty doctors. He thought people ought to go to the nearest policeman, as there would be plenty of young doctors only too willing to attend. The coroner said it struck him as being a very sad thing that when a woman was dying someone should have to run about for three or four hours to try and get a doctor. In this case, however, as they had been told by Dr. Jones, a doctor would have made no difference ; but it showed the necessity of there being some regular system with regard to night visitation. If the parish authorities appointed a man to look after their fellow creatures, it would not be a very great hardship on the ratepayers. The jury warmly concurred in the coroner's remarks, and a verdict in accordance with the medical evidence was returned.

The following vacancies are announced this week, the amount of the salary in each case being given after the name of the institution :

**Resident Medical Officers.**

Bedford General Infirmary. Surgeon—£100, board, &c.

Grove Hall Asylum, E. Medical Officer—£120, board, &c.

Brompton Consumption Hospital. House Physician.

**Non-Resident Medical Officers.**

Bristol Eye Hospital. Surgeon—£120.

The Santa Claus Society has this Christmas sent gifts of toys to the children in sixteen hospitals, and has also sent gifts of books, games, tea, etc., to the adults in the wards of St. Bartholomew's, University, German, and Orthopædic Hospitals. It is surely a very English trait, the sturdy way in which we hold Christmas festival even in hospital wards, and in spite of any amount of illness and fog and depressing circumstances generally.

The Charity Organisation Society goes steadily on its way, often held up to ridicule, yet ever doing good and useful work. It has just published its annual "black list" of institutions and individuals unfavourably known to the society, and it is strange to see placarded on this same list the names of charities which yet continue to flourish. The "Charities Register and Digest" has also been published at the price of 10s. 6d. ; it shows what a terribly complicated business it is to be wisely generous, and it also shows how great is the flood of charity—quite great enough apparently to meet all deserving cases of destitution, if it were only possible in this world to part the sheep from the goats.

Guy's kept holiday this week ; there was an entertainment on Monday in aid of the Samaritan fund, and a thoroughly good entertainment it was, and a great credit to Mr. Williams. On Tuesday the first distribution of the Butterworth medals to the nurses took place. Mr. Lushington made a little introductory speech, and Dr. Steele, in thanking Mrs. Lushington for distributing the medals, very happily remarked that it was not the intrinsic value of the medals, but the fact that their possession showed that the authorities of Guy's valued the recipients that was worth attention. An entertainment by Messrs. Craig and Campbell followed, but the programme was somewhat spoiled by the absence of half the performers through influenza. Refreshments were provided during the interval in the treasurer's dining-room, and the pictures of the founder of Guy's attracted much attention from the visitors.

A wonderful account is going the round of the press of Peter Laing, of Elgin, who last week entered on his 106th year. He is a carter and still continues to enjoy excellent health, all his faculties being unimpaired ; and any day he may be seen in the streets of Elgin in charge of his horse and cart. He is come of a family celebrated for longevity. His father died when 109 years of age, and his great grandfather, who fought under the standard of the Duke of Cumberland at the battle of Culloden, died at the remarkable age of 135. Peter lived in the time of Burns, and his memory is so retentive that he can recall an episode in the life of the Scotch national bard. He was over sixty before he was married, and since his wife died, over thirty years ago, he has been his own cook and housekeeper. He has a most robust constitution, his diet being brose, porridge, potatoes, and salt herrings ; and he confidently declares that a change in the shape of dainties would be to him worse than drugs. He was never known to have an illness with the exception of an occasional slight attack of rheumatism in one of his legs, and had never any doctor's drugs.

We have received the twenty-first annual report of Kilmarnock Infirmary. Some very interesting statistics of cases are given, including a table of diseases which proved fatal, distinguishing sexes and periods of life. The fever outbreak severely tried the resources of the infirmary last year, and the income falls far short of the outlay ; further subscriptions should be forthcoming. With regard to the fever cases, out of 58 typhoid cases only one death occurred ; and out of 106 cases of scarlet fever, only three deaths took place amongst patients who survived 48 hours. As usual, a number of children were too long in being brought to the infirmary, four of whom succumbed shortly after admission. [Particulars wrongly given last week as of Ayr Infirmary, really referred to Kilmarnock.]

The German *Medical Gazette* states that a student at Helsingborg recently sued a physician there for having hypnotised him against his will. At the hearing of the case the various witnesses for the plaintiff behaved in a most extraordinary manner, their testimony, in fact, being utterly nonsensical. The court became quite bewildered, and great confusion prevailed, till some clever (?) person discovered that all the witnesses were being hypnotised by one of the counsel. Here, then, is a new chance for the briefless barrister. Let him learn how to hypnotise his witnesses till they say just what he wants them to say, and then briefs will be showered upon the gifted young gentleman. But just fancy the poor witness, supposing the opposing counsel were both trying to hypnotise him !

In a signed article in the new *Daily Graphic*, Canon Farrar advocates more entertainments for the poor during the present dull weather. He considers the cheering influence of such entertainments is thoroughly Christian and civilising. For our part we know it is health-giving, for it is the dull and depressed that soonest fall the victims of any illness that is going. Canon Farrar refers to some old friends. He says : "The late Rev. Dr. Dawson Turner, a man of great culture and greater kindness, devoted much of his time during his later years to the hospitals—especially Westminster and Charing Cross. He got for them all sorts of illustrated newspapers and old books, and he attached great importance to the very humble service—which he assiduously performed—of going constantly from ward to ward and changing the books when they were done with. He was an excellent reciter, and he delighted in relieving the dull hours of the poor patients by reciting to them short poems, pathetic or humorous. His work is still continued by his gifted daughter, Mrs. Bewicke."

On New Year's Day the Hartley Memorial Wing of Sunderland Infirmary was opened by Alderman Preston, J.P. The visitors met in the general public-room, and punctually at half-past twelve o'clock the Mayor and Mayoress, Alderman Preston leading the way, proceeded to the dividing door between the main and the new buildings. Here the architect, Mr. Eltringham, presented Alderman Preston with a gold key with which to open the door leading to the wing. After an appropriate hymn had been sung, the Archdeacon offered up a dedicatory prayer. In the course of his speech, Alderman Preston said he found that the old infirmary cost something like £45,000, which, with the £15,000 expended on the Hartley Wing, made the noble sum of £60,000 expended upon that noble institution. That, he thought, could hardly be equalled in the North of England at all events. He congratulated the town of Sunderland upon having such a happy day, and he trusted that the New Year would be a prosperous one so far as that institution was concerned, as well as the whole of the town. The wing was inspected by all the visitors, and the proceedings terminated amidst loud applause.

